# Redox energy barrier management for efficient tin-lead perovskite solar cells

**DOI:** 10.1093/nsr/nwaf097

**Published:** 2025-03-12

**Authors:** Zhangwei He, Feng Wang, Yiman Dong, Yuling Zhang, Runnan Yu, Feng Gao, Zhan'ao Tan

**Affiliations:** Beijing Advanced Innovation Center for Soft Matter Science and Engineering, College of Materials Science and Engineering, Beijing University of Chemical Technology, Beijing 100029, China; Department of Physics, Chemistry, and Biology (IFM), Linköping University, Linköping 58183, Sweden; Beijing Advanced Innovation Center for Soft Matter Science and Engineering, College of Materials Science and Engineering, Beijing University of Chemical Technology, Beijing 100029, China; Beijing Advanced Innovation Center for Soft Matter Science and Engineering, College of Materials Science and Engineering, Beijing University of Chemical Technology, Beijing 100029, China; Beijing Advanced Innovation Center for Soft Matter Science and Engineering, College of Materials Science and Engineering, Beijing University of Chemical Technology, Beijing 100029, China; Department of Physics, Chemistry, and Biology (IFM), Linköping University, Linköping 58183, Sweden; Beijing Advanced Innovation Center for Soft Matter Science and Engineering, College of Materials Science and Engineering, Beijing University of Chemical Technology, Beijing 100029, China

**Keywords:** tandem solar cells, Sn-Pb perovskite solar cells, energy barrier, redox

## Abstract

The performance of narrow-bandgap mixed tin-lead (Sn-Pb) perovskite solar cells (PerSCs) is still limited by high defect densities due to the facile oxidation of Sn^2+^, leading to severe p-type self-doping. Here, we report a strategy of redox energy barrier managing to construct high-quality Sn-Pb perovskite, where 1,1′-bis(diphenylphosphino)ferrocene (DPPF) with a lower reaction energy barrier can protect Sn^2+^ from oxidation, where the oxidized products of DPPF tend to anchor with the Sn vacancies, effectively depressing the trap densities of perovskite. The optimized inverted PerSC reached an impressive power conversion efficiency (PCE) of 23.5% (23.38% certified) accompanied by a high open-circuit voltage of 0.89 V and a strikingly decreased energy loss of 0.36 eV. When combined with the semi-transparent wide-bandgap PerSC, the four-terminal all-perovskite tandem solar cells achieved an outstanding PCE of 26.4%.

## Teaser

This work presents a strategy to regulate the energy barrier in redox reactions for enhancing the performance of Sn-Pb single junction perovskite solar cells and tandem solar cells. The optimized inverted perovskite solar cell achieved a certified power conversion efficiency of 23.38%.

## INTRODUCTION

Organic-inorganic hybrid lead halide perovskites have revolutionized photovoltaic technology thanks to their large absorption coefficients, high mobilities and long diffusion lengths [1–3]. Nowadays, the certified power conversion efficiency (PCE) of perovskite solar cells (PerSCs) has reached high values, over 26% [4]. According to the Shockley-Queisser (S-Q) limit, lead halide perovskite absorbers with a bandgap in the range of 1.5–1.6 eV can obtain a maximum theoretical PCE of ∼30% under AM 1.5 G illumination [5]. Mixed tin-lead (Sn-Pb) perovskites with narrow bandgaps (1.2–1.4 eV) have shown even greater potential in boosting the maximum theoretical PCE to values over 30%. Moreover, Sn-Pb perovskites are promising candidates in multi-junction all-perovskite tandem solar cells, with the potential to surpass the S-Q efficiency limitations [6–9]. However, the photovoltaic performance of Sn-Pb PerSCs remains inferior to their pure-Pb counterparts, limited by the high trap densities arising from the prone-to-oxidation nature of Sn^2+^ [10]. Such an oxidation process can occur during precursor preparation, film manufacture, device operation and storage. This results in p-type self-doping, causing severe energy loss and poor stability for Sn-Pb PerSCs [11–14].

Dedicated efforts have been made to address these issues through compositional engineering, interface passivation and additive engineering [15–18]. On the one hand, the intrinsic defects of Sn-Pb perovskites can be partially healed through passivation. Amine molecules and guanidinium have emerged as two of the most promising passivators for optimizing Sn-Pb PerSCs. By directly incorporating phenethylammonium ligands into the antisolvent, defect passivation is achieved not only on the surface of the perovskite film but also at the grain boundaries, leading to improved efficiency and operational stability in Sn-Pb PerSCs [19]. Furthermore, a selective targeting anchor approach has been developed by utilizing phenethylammonium iodide and ethylenediamine diiodide as co-modifiers, which can attach to Pb- and Sn-associated active sites and manage dual-metal traps, resulting in enhanced open-circuit voltage (*V*_OC_) [20]. The addition of guanidinium thiocyanate into a Sn-Pb perovskite precursor improved the structural and optoelectronic properties, leading to reduced energetic disorder with decreased defect densities [21]. On the other hand, antioxidants and reducing agents have been introduced into the perovskite precursor as additives to inhibit the oxidation of Sn^2+^ [22–24]. Recently, caffeic acid with hydroxyl functional groups has been applied to suppress Sn^2+^ oxidation, resulting in restrained background carriers and hole trap densities [25]. A reducing agent, 4-hydrazinobenzoic acid, containing hydrazine groups, can form an amorphous complex along with SnF_2_ to suppress the oxidation of Sn^2+^, enhancing carrier separation and suppressing charge recombination [26]. Most additives designed to inhibit the oxidation of Sn^2+^ usually function by reducing Sn^4+^ to Sn^2+^, thus achieving a restorative effect. However, a potential drawback of this approach is that the oxidation by-products formed may negatively impact device performance. A potential approach to solving this challenge is to use additives as sacrificial agents to directly protect Sn^2+^ from oxidation.

In this work, a strategy of redox energy barrier manipulation was applied to inhibit Sn^2+^ oxidation by incorporating an organometallic complex, 1,1′-bis(diphenylphosphino)ferrocene (DPPF), into the Sn-Pb perovskites. We first revealed that DPPF inhibits Sn^2+^ oxidation by preferentially reacting with oxygen molecules, thereby protecting Sn^2+^ from oxidation. In addition, the oxidation products of DPPF, namely DPPFO and DPPFO_2_, can anchor through Pb^2+^ and Sn^2+^ through lone pair electrons on the oxygen of –P=O groups to effectively suppress the formation of excessive Sn vacancies and reduce trap densities. Benefitting from the high-quality and stable perovskite films with enhanced charge extraction and decreased non-radiative recombination losses, the DPPF-modified Sn-Pb PerSC delivers a remarkably high PCE of 23.5% (23.38% certified) with a *V*_OC_ of 0.89 V and an outstanding low energy loss (*E*_loss_) of 0.36 eV. When combining the DPPF-modified bottom Sn-Pb PerSC with a semi-transparent wide-bandgap top PerSC, the 4-terminal all-perovskite tandem solar cell delivers an optimal PCE of 26.4%.

## RESULTS AND DISCUSSION

### Antioxidant behavior and mechanism of DPPF

**Figure 1.**
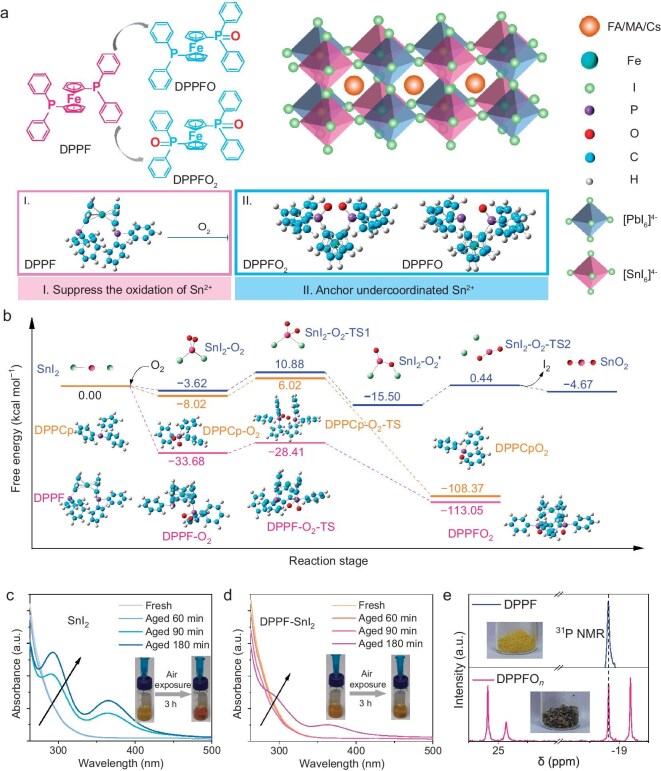
DFT analysis of the antioxidant mechanism and suppression of Sn^2+^ oxidation. (a) Schematic illustration of the suppression of Sn^2+^ oxidation by DPPF and defects passivation by the oxidation products of DPPF. (b) DFT-computed reaction free energy profiles for the oxidation of SnI_2_, DPPCp and DPPF. Optimized geometries of the pristine molecules, intermediate and TS structures are given. UV-Vis absorption spectra of (c) SnI_2_ and (d) DPPF-SnI_2_ dissolved in DMF solution. The insets are photographs of the color change of SnI_2_ precursor solutions with and without DPPF additive after 3 h of exposure to ambient air. (e) ^31^P NMR spectra and photographs of DPPF and DPPFO*_n_*.

DPPF with strong redox reactions with oxygen molecules was chosen to prevent the Sn^2+^ oxidation existing in the perovskite. In addition, the oxidized products of DPPF, DPPFO and DPPFO_2_ can chelate with uncoordinated Pb^2+^ and Sn^2+^ through lone pair electrons on oxygen from –P=O groups to passivate defects (Fig. 1a). To explore the mechanism behind the inhibition of Sn^2+^ oxidation, both theoretical and experimental analyses were conducted. To confirm the pathway of oxidation reaction, we performed density functional theory (DFT) calculations (Fig. 1b). We define the transition state as TS during the DFT-computed reaction process. The reaction begins with the DPPF, which reacts with O_2_ to form intermediate DPPF-O_2_ (−33.68 kcal/mol). Next, DPPFO_2_ is formed from DPPF-O_2_ via DPPF-O_2_-TS with an energy of 5.27 kcal/mol. For SnI_2_, it undergoes oxidative addition to generate SnI_2_-O_2_, which then converts to SnI_2_-O_2_-TS1 with an energy of 14.5 kcal/mol, followed by the formation of SnI_2_-O_2_^’^. Finally, the elimination of I_2_ yields the final product SnO_2_ with a high energy of −4.67 kcal/mol. We also calculated the oxidation process between ligands of DPPF, cyclopentadien-5-yldiphenylphosphine (DPPCp) and O_2_ via intermediate DPPCp-O_2_ and DPPCp-O_2_-TS, which indicates that the energy is −108.37 kcal/mol, higher than that of DPPF (−113.05 kcal/mol). These results suggest that both SnI_2_ and DPPCp have a lower tendency to produce oxidation products compared to DPPF, indicating that the DPPF can effectively block the redox reactions between Sn^2+^ and oxygen molecules. Furthermore, differential scanning calorimetry was employed to assess the oxidation induction behavior of DPPF and SnI_2_. The results (Fig. S1) indicate that the onset of oxidation for SnI_2_ occurs later than that for DPPF, demonstrating that DPPF reacts with oxygen preferentially over SnI_2_. Meanwhile, we performed cyclic voltammetry characterization to analyze their redox potentials (Fig. S2). In the cyclic voltammetry curves, obvious oxidation and reduction peaks are observed, allowing for the calculation of the redox potential (*E*_1/2_) by averaging the cathode potential (*E*_c_) and anode potential (*E*_a_) [27]. For SnI_2_, the calculated *E*_1/2_ is −0.509 V. Compared to the SnI_2_, the *E*_1/2_ of the DPPF decreased to −0.849 V. The lower redox potential than Sn^2+^ indicates that DPPF reacts with oxygen more easily, possessing the ability to protect Sn^2+^ from being oxidized. Diverging from conventional strategies where additives primarily function as Sn^4+^ reductants, DPPF prevents Sn^2+^ oxidation through preferential binding with oxygen molecules, as evidenced by its significantly lower oxygen scavenging energy barrier.

The theoretical results prompt us to examine the protection capacity of DPPF. Figure 1c illustrates that the SnI_2_ in *N,N*-dimethylformamide (DMF) solution shows a color change from yellow to deep red after continuous exposure to ambient air (the specific color change process is displayed in the movie S1), which usually represents the oxidation of Sn^2+^ to Sn^4+^ [28]. The ultraviolet-visible (UV-Vis) absorption spectra of the solution further confirmed the Sn^2+^ oxidation progress. Considering that the oxidation from Sn^2+^ to Sn^4+^ has priority over that from I^−^ to complex anion tri-iodide I_3_^−^ (a continued exergonic equilibrium gives as follows, I_2_ + I^−^ ⇄ I_3_^−^), we can evaluate the oxidation process of Sn^2+^ by monitoring the occurrence time of the I_3_^−^ characteristic peaks at 293 and 365 nm [29,30]. The pure SnI_2_ solution exhibits noticeable I_3_^−^ characteristic signals after exposure to the air for 90 min. In contrast, there is only a tiny I_3_^−^ signal in the DPPF-SnI_2_ blend solution after aging for 180 min (Fig. 1d). As shown in the inset photos, the SnI_2_ solution with DPPF still remains yellow after aging in the air for the same time, which agrees well with the UV-Vis results. Consequently, it can be deduced that DPPF can protect Sn^2+^ from oxidation. To analyze the oxidation products of DPPF, the reaction mixtures were stirred and purged with oxygen for 12 h. The pure yellow DPPF sample turned into gray powder after oxidation (Fig. 1e). We further conducted phosphorus nuclear magnetic resonance (^31^P NMR) spectroscopy to analyze the oxidation products. It was found that only one signal appeared at −18.82 ppm in the spectrum of DPPF, while three new peaks appeared in the DPPF oxidation products. The characteristic peaks at 24.86 and −19.19 ppm were ascribed to DPPFO, and the peak at 25.18 ppm was attributed to DPPFO_2_ [31–34]. The oxidation products of DPPF hereafter will be denoted as ‘DPPFO*_n_*’. Interestingly, when DPPF powders were added to the oxidized SnI_2_ solution, the reddish-brown solution became orange again, proving that Sn^4+^ was reduced to Sn^2+^ in the presence of DPPF (Fig. S3 and Movie S2). As shown in Fig. S4, compared to the oxidized SnI_2_ solution without any characteristic peaks, four new peaks appeared after the addition of DPPF powders, which we attribute to the DPPFO*_n_* present in the solution. These phenomena fully suggest that DPPF could suppress the oxidation of Sn^2+^ and promote the reduction of Sn^4+^. This can be further confirmed by X-ray photoelectron spectroscopy (XPS) characterizations to analyze the antioxidation effect of DPPF in Sn-Pb perovskite films. The Sn 3d XPS spectra of both perovskite films are depicted in Figs S5 and S6. The Sn 3d_5/2_ peaks are divided into two typical peaks, which are associated with Sn^4+^ and Sn^2+^, respectively [35]. Fig. S5 shows that fresh perovskite film with or without DPPF modification exhibited a similar proportion of Sn^2+^ and Sn^4+^. However, after exposure to air for 15 min, the DPPF-modified perovskite film shows a greater reduction of Sn^4+^ content than the pristine film (Fig. S6), confirming the ability of DPPF to suppress Sn^2+^ oxidation.

### Coordination between Sn^2+^ and DPPF oxidation products

**Figure 2.**
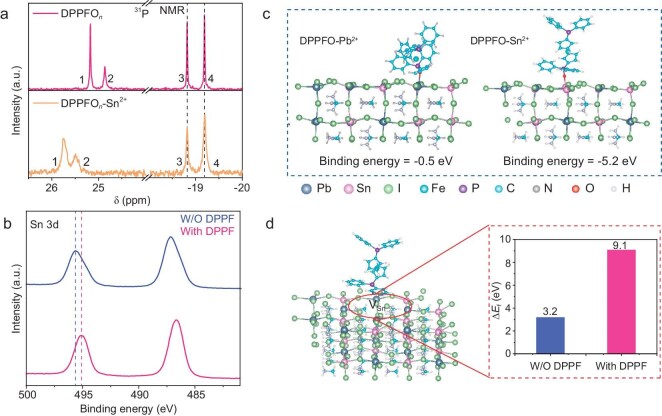
Coordination behavior of DPPF oxidation products on Sn^2+^. (a) ^31^P NMR spectra of DPPFO*_n_* and DPPFO*_n_*-Sn^2+^. 1 refers to the P=O peak from DPPFO_2_, 2 and 4 refer to the P=O peak from DPPFO, and 3 refers to the P peak from DPPF. (b) XPS spectra of Sn 3d in perovskite film with and without DPPF modification. (c) Optimized models and related binding energies of DPPFO coordinate with Pb^2+^ or Sn^2+^ in Sn-Pb perovskite. (d) Defect formation energies (Δ*E*_f_) of V_Sn_ with and without DPPF modification.

Next, we focus on understanding the interaction between perovskite and DPPFO*_n_* in Pb-Sn perovskite. ^31^P NMR spectroscopy was conducted to determine the coordination interactions between DPPFO*_n_* and Sn^2+^ in perovskite. As shown in Fig. 2a, after adding DPPF into perovskite precursors, there is no shift in the peak position of P from DPPF (−18.82 ppm) and DPPFO (−19.19 ppm), indicating that the P sites in the DPPF and DPPFO are not involved in the coordination reaction. The peak position of P=O from DPPFO and DPPFO_2_ exhibits an apparent downfield shift from 24.86 and 25.17 ppm to 25.47 and 25.73 ppm, respectively, suggesting the change of chemical environment of the phosphorous nucleus [36], possibly caused by the sharing of a lone pair electron of oxygen in P=O with Sn^2+^. For a better understanding of the coordination interactions from the view of surface chemistry, we carried out XPS measurements on both films (Fig. 2b). The high-resolution XPS spectra of Sn 3d in the perovskite film exhibit two prominent peaks at 495.6 and 487.2 eV, which correspond to Sn 3d_3/2_ and Sn 3d_5/2_, respectively [37]. After the DPPF modification, these peaks shift to the lower binding energies of 495.1 and 486.7 eV, respectively, demonstrating the chemical interactions between Sn^2+^ and P=O bonds in DPPFO*_n_*. Similarly, the XPS spectra of Pb 4f also exhibit the same trend. The peaks attributed to Pb 4f_5/2_ and 4f_7/2_ downshifted from 143.0 and 138.2 eV to 142.8 and 138.0 eV, respectively (Fig. S7). Additionally, the O 1s spectrum from XPS also corroborates this conclusion (Fig. S8). In the DPPFO*_n_* sample, the O 1s peak exhibits a distinct signal with a binding energy of 531.7 eV, indicating that oxygen primarily exists in the form of P=O bonds. After the introduction of Sn^2+^ into the DPPFO*_n_* sample, the peak shifted to a higher binding energy (532.4 eV), indicating the interaction with Sn^2+^ with the formation of P=O···Sn coordination bond. The interaction can be further confirmed by Fourier transform infrared (FTIR) spectroscopy (Fig. S9), where the stretching vibration peak of N–H shifts from 3398 cm^−1^ for the control film to 3393 cm^−1^ for the DPPF-modified film, denoting a significant interaction between the DPPFO*_n_* and perovskite. We further conducted DFT calculations to evaluate the binding energy of DPPFO on the perovskite surface (Fig. 2c). The binding energy of DPPFO with Sn^2+^ and Pb^2+^ is −5.2 eV and −0.5 eV, respectively. In addition, the passivation strategy is found to increase the defect formation energy of the V_Sn_ from 3.2 eV (without modification) to 9.1 eV (with DPPFO), reducing the number of vacancies (Fig. 2d).

X-ray diffraction (XRD) characterization was conducted to analyze the crystallinity of the perovskite films with and without DPPF modification. As shown in Fig. S10, the DPPF-modified film exhibits no new peaks or peak shifts compared with the control film, indicating that the DPPFO*_n_* exists on the crystal boundaries rather than within the crystal lattice. In addition, DPPF-modified film showed a reduction in the full-width half-maximum (FWHM) values from 0.09° to 0.08° for the (100) peak, confirming that DPPFO*_n_* coordinated with perovskite to enlarge grain size (Fig. S11). Furthermore, the (100) orientation peak of the target perovskite film is stronger than that of the control one, indicating that the DPPF-modified perovskite film exhibits stronger crystallinity. To further unveil the comprehensive impacts of DPPF on perovskite film quality, top-view scanning electron microscopy (SEM) and atomic force microscopy (AFM) were conducted. As shown in the SEM images (Fig. S12), the grain size enlarged after the DPPF modification. The AFM images of the perovskite films with and without DPPF modification are shown in Fig. S13, respectively. The root-mean-square roughness (*R*_q_) of the DPPF-modified perovskite film (49.1 nm) is larger than that of the control perovskite film (37.3 nm), possibly due to the larger grains of DPPF-modified perovskite film.

### Performance of single-junction device and optoelectronic characterization

**Figure 3.**
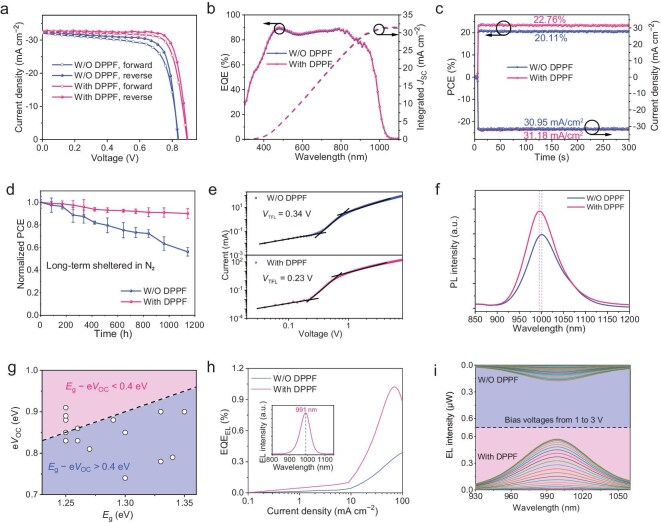
Photovoltaic performance of single-junction solar cells and photoelectronic characterizations. (a) The *J*-*V* curves of the PerSCs with and without DPPF modification. Both the reverse and forward scans are included. (b) The corresponding EQE curves. (c) SPO at the maximum power point (MPP) for the PerSCs with and without DPPF modification. (d) Long-term stability testing of unencapsulated PerSCs with and without DPPF modification. The devices are sheltered in an N_2_ atmosphere at room temperature. (e) SCLC plots of electron-only devices. (f) PL spectra of Sn-Pb perovskite films with and without DPPF modification. (g) Plots of e*V*_OC_ against *E*_g_ for Sn-Pb PerSCs reported in the literature so far, with all data summarized in Table S4. (h) EQE_EL_ values of the PerSCs with and without DPPF modification under different current densities. The inset shows EL spectra of the DPPF-modified PerSC. (i) EL spectra of devices with and without DPPF modification under various bias voltages.

Inverted devices with a configuration of indium tin oxide (ITO)/poly(3,4-ethylenedioxythiophene): polystyrene sulfonate (PEDOT:PSS)/perovskite/EDAI_2_/C60/bathocuproine (BCP)/Ag were fabricated to investigate the effect of DPPF modification on photovoltaic performance. The concentration of DPPF was optimized as 0.3 mg/mL (Fig. S14), and the detailed photovoltaic parameters are displayed in Table S1. Subsequently, we further optimized the device performance by post-treatment processing (Fig. S15). Table S2 summarizes the corresponding photovoltaics parameters. Figure 3a presents the current density-voltage (*J*-*V*) curves measured under AM 1.5 G illumination at 100 mW cm^−2^, and the relevant photovoltaic parameters are summarized in Table 1. The PerSC without DPPF modification shows a *V*_OC_ of 0.84 V, a short-circuit current density (*J*_SC_) of 32.5 mA cm^−2^ and a fill factor (FF) of 75.1%, yielding a PCE of 20.5%. In contrast, the DPPF-modified PerSC delivers a maximum PCE of 23.5%, with a much higher *V*_OC_ of 0.89 V, a *J*_SC_ of 32.7 mA cm^−2^ and an FF of 80.7%. Notably, our Sn-Pb PerSC has achieved a certified PCE of 23.38% (Fig. S16). The hysteresis behavior in narrow-bandgap PerSCs may be associated with voltage scan protocols or specific device-related deficiencies. Figure 3b shows the external quantum efficiency (EQE) spectra of the control and DPPF-modified PerSCs, and the integrated current densities of the corresponding PerSCs are 31.3 and 31.6 mA cm^−2^, respectively, which match well with the values measured by the *J*-*V* curves. As shown in Fig. S17, the bandgap of both films estimated from the derivative of the corresponding EQE spectra is ∼1.25 eV. Figure S18 presents the statistics and the distribution of photovoltaic parameters of 20 independent PerSCs for different batches. The values of all average photovoltaic parameters for DPPF-modified PerSCs are superior to that of the control PerSCs, confirming the excellent repeatability of the DPPF modification strategy. Beyond improved photovoltaic performance, the DPPF-modified PerSCs also show enhanced stability. As plotted in Fig. 3c, the stable power output (SPO) of the DPPF-modified PerSC exhibits a stable PCE of 22.76% at the fixed maximum power point bias of 0.73 V under continuous illumination for 300 s, much higher than that of the control device (20.11% at 0.65 bias). In addition, as illustrated in Fig. 3d, after being stored in a nitrogen atmosphere for over 1100 h, the DPPF-modified PerSCs can maintain 90% of their original efficiency, while the control PerSCs only retain 56% of their initial PCE. As depicted in Fig. S19, we also assessed the operational stability of the PerSCs. The PerSCs without DPPF modification demonstrated significant performance degradation, with the PCE dropping to 80% of the initial value after 120 hours of storage. In contrast, the DPPF-modified PerSCs exhibited enhanced stability, retaining 90% of their original PCE even after 380 hours of illumination. The strong *in situ* anchoring effect of DPPFO*_n_* on perovskite is the critical element for device stability improvement.

**Table 1.** Photovoltaic parameters of the PerSCs with or without DPPF modification.

Condition	Scan direction	*V*_OC_ (V)	*J*_SC_ (mA cm^−2^)	FF (%)	PCE (%)^a^
W/O DPPF	Forward	0.84 (0.82 ± 0.02)	32.1 (31.7 ± 0.4)	71.4 (70.0 ± 1.0)	19.3 (18.2 ± 0.3)
	Reverse	0.84 (0.82 ± 0.02)	32.5 (32.5 ± 0.1)	75.1 (74.1 ± 0.8)	20.5 (19.8 ± 0.4)
With DPPF	Forward	0.89 (0.87 ± 0.02)	32.4 (32.0 ± 0.3)	77.8 (76.3 ± 0.8)	22.5 (21.2 ± 0.2)
	Reverse	0.89 (0.88 ± 0.01)	32.7 (32.6 ± 0.1)	80.7 (79.2 ± 0.7)	23.5 (22.7 ± 0.3)
With DPPF^b^	Reverse	0.887	32.625	80.76	23.38

a The average parameters with standard deviations calculated from 20 devices are listed in parentheses. ^b^Certified result from Institute of Electrical Engineering, Chinese Academy of Sciences.

Space-charge-limited-current (SCLC) measurements based on the electron-only devices with a structure of ITO/SnO_2_/perovskite/C60/BCP/Ag and the hole-only devices with a structure of ITO/PEDOT:PSS/perovskite/Spiro-MeOTAD/MoO_3_/Ag were performed under dark conditions to calculate the trap density of devices with and without DPPF modification (Table S3). For the electron-only devices, the trap-filled limit voltage (*V*_TFL_) of the control device is 0.34 V and is decreased to 0.23 V after DPPF modification (Fig. 3e). The calculated electron trap density (*N*_t_) of the control and DPPF-modified devices is 1.7 × 10^15^ and 1.2 × 10^15^ cm^−3^, respectively. As depicted in Fig. S20, for the hole-only devices, the *V*_TFL_ of the DPPF-modified device (0.46 V) is also lower than that of the device without DPPF modification (0.52 V). The corresponding hole *N*_t_ of the DPPF-modified devices (2.4 × 10^15^ cm^−3^) was decreased compared to the control one (2.8 × 10^15^ cm^−3^). These results verify the decreased trap density as well as reduced carrier recombination in the DPPF-modified PerSCs [38]. In addition, the *V*_OC_ under different light intensities (*P*_light_) is measured to attain more insights into the carrier recombination behavior in both devices. The correlation between *V*_OC_ and logarithmic *P*_light_ is extracted by the formula of *V*_OC_ ∝ *nk*_B_*T*ln(*P*_light_)/e, where *n, k*_B_ and *T* represent the ideality factor, the Boltzmann constant and absolute temperature, respectively. As illustrated in Fig. S21, the *n* value of the device without DPPF modification is 1.84. After being modified by DPPF, a smaller value of 1.48 is achieved owing to the repressed trap-assisted recombination and prolonged lifetimes [39]. Then, steady-state photoluminescence (PL) is conducted to investigate the influence of DPPF modification on carrier dynamics. As plotted in Fig. 3f, the PL intensity of the DPPF-modified perovskite film is stronger than that of the control film. Moreover, the peak center shifts slightly from 1000 to 995 nm after DPPF modification. The improved PL intensity and blue-shifted PL peak center is consistent with the inhibited non-radiative recombination and reduced trap state densities in the film upon DPPF modification.

With the DPPF modification strategy, we successfully fabricated efficient and reproducible Sn-Pb PerSCs with high *V*_OC_. Figure 3g presents plots of e*V*_OC_ against *E*_g_ for Sn-Pb PerSCs reported so far, and the detailed parameters are summarized in Table S4. The energy loss (*E*_loss_) can be calculated by the formula *E*_loss_ = *E*_g_ − e*V*_OC_, where e is the elementary charge. The *E*_loss_ values for most Sn-Pb PerSCs lie above 0.4 eV. It is worth noting that the *E*_loss_ (0.36 eV) obtained in our work is one of the lowest among the Sn-Pb PerSCs reported so far. The *E*_loss_ is closely associated with the energy disorder and defect states that create non-radiative recombination energy loss paths and therefore decrease *V*_OC_ [40–42]. To quantitatively analyze the reasons for *E*_loss_ improvement, we calculated the non-radiative recombination *V*_OC_ loss (∆$V_{{\mathrm{OC}}}^{{\mathrm{non - rad}}}$) of the PerSCs with and without DPPF modification, which can be obtained by the equation ∆$V_{{\mathrm{OC}}}^{{\mathrm{non - rad}}}$ = −$\frac{{{k_B}T}}{q}$ln(EQE_EL_), where EQE_EL_ is the EQE of electroluminescence (EL). The EQE_EL_ was measured by operating the PerSCs as light-emitting diodes (LEDs), as illustrated in Fig. 3h. For the PerSC without DPPF modification, the EQE_EL_ value of 0.20% can be acquired with the EL peak located at 991 nm and the corresponding ∆$V_{{\mathrm{OC}}}^{{\mathrm{non - rad}}}$ was calculated to be 161 mV. In comparison, the DPPF-modified PerSC achieved an EQE_EL_ of 0.66%, leading to a ∆$V_{{\mathrm{OC}}}^{{\mathrm{non - rad}}}$ decrease to 130 mV. The relatively lower ∆$V_{{\mathrm{OC}}}^{{\mathrm{non - rad}}}$ value confirms the superior passivation effect, contributing to the realization of a high *V*_OC_. We further compared the EL emission intensity of the device by conducting EL measurements on both PerSCs (Fig. 3i). Under any current injection condition, the DPPF-modified PerSCs showed a relatively higher EL emission intensity than the control device [43,44]. To assess the influence of the DPPF modification on the suppression of the trap density, we calculated the Urbach energy (*E*_u_) of corresponding perovskite films by fitting the slope in the long-wavelength edge of the experimental EQE spectra [45]. Compared to the control film, the DPPF-modified film exhibits a decreased *E*_u_ of 23 meV from 27 meV (Fig. S22), indicating a prolonged carrier lifetime and a reduced trap density [46]. Moreover, a lower *E*_u_ indicates a higher phase or structural stability of the perovskite film, as well as a lower *E*_loss_ [47].

Structural analysis reveals that the ferrocene moieties in DPPF, characterized by their electron-rich nature and extensive π-electron delocalization, facilitate rapid electron transport in perovskite. To confirm the impact of the strategy on charge transfer dynamics and recombination process in PerSCs, a range of optoelectronic measurements were conducted, including dark *J*-*V* characterizations and transient photo-current decay (TPC). As illustrated in Fig. S23, the dark *J*-*V* curve of the DPPF-modified device shows a reduced leakage current compared to the control device, indicating the decreased trap density and inhibited carrier recombination, which contributes to the improved *V*_OC_ and FF. TPC measurements (Fig. S24) show that the carrier collection lifetime of the DPPF-modified PerSCs is reduced, demonstrating that the charge transfer is considerably accelerated. Nyquist plots of devices with and without DPPF modification (Fig. S25) are evaluated without bias voltage under AM 1.5 G illumination. By fitting the electrochemical impedance spectroscopy (EIS) results employing the equivalent model containing a series resistance (*R*_s_) and a charge transport resistance (*R*_ct_), it was found that the *R*_ct_ of the device from the DPPF-modified device (851 Ω) is significantly lower than that from the control device (1196 Ω), which proves the suppressed charge recombination and thereby the improved charge extraction in PerSCs. We also present a light-dependent EIS measurement in open-circuit conditions (Fig. S26). The charge transfer resistance exhibits a significant reduction with increasing light intensity, demonstrating enhanced interfacial charge transfer efficiency that facilitates improved charge transport characteristics.

### Photovoltaic performance of tandem solar cells

**Figure 4.**
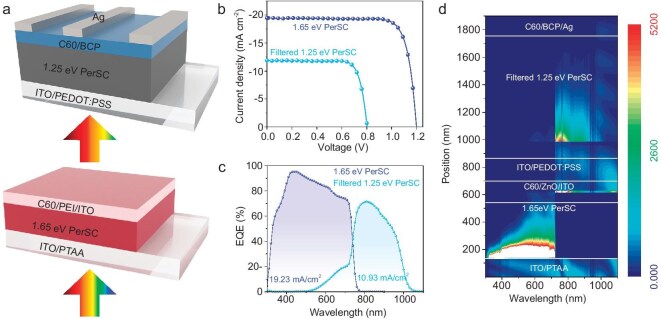
Photovoltaic performance of tandem solar cells. (a) Device architecture of all-perovskite 4T tandem solar cells. (b) *J*-*V* curves of the single-junction semi-transparent top PerSC and the filtered Sn-Pb bottom PerSC under AM 1.5 G illumination measured in the reverse direction. (c) EQE spectra of the all-perovskite 4T tandem solar cells. (d) The distributions of photon absorptions in tandem solar cells, simulated by the transfer matrix method.

Ultimately, the 4-terminal (4T) all-perovskite tandem solar cells are mechanically stacked using a bottom Sn-Pb PerSC with a bandgap of ∼1.25 eV and a semi-transparent (bandgap ≈ 1.65 eV) top PerSC (Fig. 4a). The semi-transparent top PerSC has an inverted structure of ITO/poly[bis(4-phenyl)(2,4,6-triMethylphenyl)aMine] (PTAA)/FA_0.78_Cs_0.22_Pb(I_0.82_Br_0.15_Cl_0.03_)_3_/C60/polyethyleneimine (PEI)/ITO. The *J*-*V* curves of single-junction semi-transparent top PerSC and bottom Sn-Pb PerSC with the semi-transparent top subcell as the optical filter are depicted in Fig. 4b. Their corresponding photovoltaic parameters are summarized in Table 2. The semi-transparent top PerSC has a PCE of 18.8%, while the filtered bottom Sn-Pb PerSC delivers a PCE of 7.6%. As a consequence, the 4T tandem cell achieves a PCE of 26.4%, calculated by summing the efficiency of the semi-transparent top PerSC and filtered bottom Sn-Pb PerSC. As shown in Fig. 4c, the integrated *J*_SC_ calculated based on the obtained EQE for the semi-transparent top PerSC and the filtered bottom Sn-Pb PerSC are 19.23 and 10.93 mA cm^−2^, respectively, which align closely with the *J*_SC_ measured from the *J*-*V* curve. To further analyze the light field distribution, optical transfer matrix formalism simulation models were established (Fig. 4d). The optical constants (refraction index and absorption coefficient) of wide-bandgap perovskite and Sn-Pb perovskite are illustrated in Fig. S27. The simulation results are consistent with the EQE spectra.

**Table 2.** Photovoltaic parameters of perovskite single-junction and all-perovskite 4T tandem solar cells.

Device	Scan direction	*V*_OC_ (V)	*J*_SC_ (mA cm^−2^)	FF (%)	PCE (%)
1.65-eV PerSC (semi-transparent)	Forward	1.19	19.2	79.1	18.1
	Reverse	1.20	19.5	80.4	18.8
Filtered 1.25-eV PerSC	Forward	0.80	11.6	75.6	7.0
	Reverse	0.80	11.9	79.3	7.6
4T tandem	Forward	-	-	-	25.1
	Reverse	-	-	-	26.4

## CONCLUSION

In summary, we applied a redox energy management strategy to fabricate high-quality Sn-Pb perovskite layers, enabling efficient single-junction photovoltaics and all-perovskite tandem solar cells. Theoretical calculations proved that DPPF exhibits a higher reactivity with oxygen molecules than Sn^2+^, indicating the role of DPPF in protecting Sn^2+^ from being oxidized rather than only reducing Sn^4+^. Additionally, the oxidation products, DPPFO and DPPFO_2_, can effectively anchor uncoordinated Sn^2+^ and Pb^2+^ through –P=O groups, leading to increased defect formation energies and decreased trap densities. As a result, the DPPF-modified devices simultaneously achieved lower *E*_loss_, inhibited charge recombination and improved carrier extraction. The optimized device achieves an energy loss as low as 0.36 eV and an excellent PCE of 23.5% (23.38% certified). Furthermore, by combining the semi-transparent top PerSC, a 4T all-perovskite tandem solar cell successfully delivered a high PCE of 26.4%. Our results reveal antioxidation-associated mechanisms and emphasize the importance of guiding additive engineering through a holistic perspective, paving the way for further improvements to the performance of single-junction Sn-Pb PerSCs and tandem solar cells.

## Supplementary Material

nwaf097_Supplemental_Files
