# Supplementary material for: Redox energy barrier management for efficient tin-lead perovskite solar cells
Source: Natl Sci Rev. 2025 Mar 12;12(5):nwaf097. doi: 10.1093/nsr/nwaf097 (PMC11980978; doi:10.1093/nsr/nwaf097)
Supplement: nwaf097_Supplemental_Files [file nwaf097_supplemental_files.zip › Supplementary_Materials.pdf]

Supplementary Materials

Redox Energy Barrier Management for Efficient Tin-Lead Perovskite Solar Cells

Zhangwei He[1,†], Feng Wang[2,†], Yiman Dong[1], Yuling Zhang[1], Runnan Yu[1,*], Feng Gao[2,*] and Zhan'ao Tan[1,*]

[1]*Beijing Advanced Innovation Center for Soft Matter Science and Engineering, College of Materials Science and Engineerin, Beijing University of Chemical Technology, Beijing, 100029, China*

[2]*Department of Physics, Chemistry, and Biology (IFM), Linköping University, Linköping, Sweden*

*Correspondence to: yurunnan@mail.buct.edu.cn; feng.gao@liu.se; tanzhanao@mail.buct.edu.cn

[†]These authors contributed equally to this work

Materials and Methods

Materials

Lead iodide (PbI_2, 99.99%), tin (II) fluoride (SnF_2, 99.999%), tin (II) iodide (SnI_2, beads, 99.999%), and 2,9-Dimethyl-4,7-diphenyl-1,10-phenanthroline (BCP, 99.5%) were purchased from Advanced Election Technology. Ammonium thiocyanate (NH_4SCN, 99.99%) was purchased from Aladdin. Organic salt of formamidinium iodide (FAI) was purchased from Greatcell Solar Materials (Australia). Poly[bis(4-phenyl)(2,4,6-triMethylphenyl)aMine] (PTAA), lead chloride ($PbCl_2$, ≥99.5%), methylamine chloride (MACl), lead bromide ($PbBr_2$, ≥99.5%), fullerene (C60), cesium iodide (CsI, 99.99%) and methylammounium iodide (MAI, ≥99.5%) were purchased from Xi'an Polymer Light Technology Corp. Ethane-1,2-diammonium iodide (ethylenediammonium diiodide, $EDAI_2$, 98%) was purchased from Sigma-Aldrich Co., Ltd. Poly(3,4-ethylenedioxythiophene): poly(styrene sulfonate) (PEDOT:PSS) aqueous solution (Clevious PVP AI 4083) was purchased from Heraeus Co., Ltd. N, N-dimethylformamide (DMF, 99.8%), dimethyl sulfoxide (DMSO, >99.7＋%), isopropanol (IPA, 99.8%), toluene, Ethyl acetate (EA, 99.8%) and chlorobenzene (CB, 99.8%) were purchased from Acros Organics. 1,1'-Bis(diphenylphosphino)ferrocene (DPPF, 98%) was purchased from Energy Chemical. All chemicals were used as received without further purification.

Narrow-bandgap perovskite solar cells fabrication

In this work, narrow-bandgap perovskite solar cells were fabricated with a configuration of ITO/PEDOT:PSS/Perovskite/C60/BCP/Ag. The devices were fabricated as the following process. The ITO substrates were sequentially washed by detergent, ultrapure water, and ethanol through ultrasonic treatment in cleanser essence for 15 min. Then the ITO substrates were dried in an oven at 65 ℃ for 15 min and treated by ultraviolet-ozone (UVO) for 15 min. Then ITO substrates were spin-coated with PEDOT:PSS at 3000 rpm for 30 s, followed by baking at 150 °C for 15 min. The perovskite precursor solution (1.8 M) composed of CsI, MAI, FAI, PbI_2, SnI_2, SnF_2 and NH_4SCN was dissolved in a mixed solvent (DMF/DMSO

= 4:1) with a chemical formula of $Cs_{0.1}FA_{0.6}MA_{0.3}Sn_{0.5}Pb_{0.5}I_3$. The perovskite precursor solution was spin-coated onto the substrate at 1000 rpm for 10 s, and 4000 rpm for 40 s. 150 μL CB antisolvent was dropped on top of the spinning substrates during the second stage at 20 s before the end of the procedure. Sequentially, the perovskite film was annealed at 100 °C for 10 min and 65 °C for 10 min. The perovskite solutions with 0.1 mg/mL, 0.3 mg/mL and 0.5 mg/mL DPPF were prepared by mixing control perovskite solution and DPPF. Then, $EDAI_2$ solution was spin-coated on perovskite films at 4000 rpm for 20 s. Finally, 30 nm C60, 8 nm BCP and 100 nm Ag were thermally deposited by evaporation.

Wide-bandgap semi-transparent perovskite solar cells fabrication

In this work, wide-bandgap semi-transparent perovskite solar cells were fabricated with a configuration of ITO/PTAA/Perovskite/C60/ZnO/ITO. The devices were fabricated as the following process. The ITO substrates were sequentially washed by detergent, ultrapure water, and ethanol through ultrasonic treatment in cleanser essence for 15 min. Then the ITO substrates were dried in an oven at 65 °C for 15 min and treated by ultraviolet-ozone (UVO) for 15 min. Then ITO substrates were spin-coated with PTAA at 3000 rpm for 30 s, followed by baking at 150 °C for 15 min. The perovskite precursor solution (1.4 M) composed of CsI, FAI, PbI_2, $PbBr_2$, $PbCl_2$ and MACl was dissolved in a mixed solvent (DMF/DMSO = 3:1) with a chemical formula of $[(Cs_{0.22}FA_{0.78}Pb(I_{0.85}Br_{0.15})_3]_{0.97}(MAPbCl_3)_{0.03}$. The perovskite precursor solution was spin-coated onto the substrate at 5000 rpm for 50 s. 300 μL EA antisolvent was dropped on top of the spinning substrates during the second stage at 25 s before the end of the procedure. Sequentially, the perovskite film was annealed at 100 °C for 30 min. Then, 15 nm C60 was thermally deposited by evaporation. Later, 1 mg/ml PEI solution (dissolved in IPA) was spin-coated on the top of C60 layer at 5000 rpm for 30 s. Next, the top transparent ITO electrode of 150 nm was sputtered through a shadow mask by magnetron sputtering with a radio frequency power of 100 W. Finally, the 100 nm Ag finger was deposited on the edge of the active area of the ITO electrode.

Characterizations

^{31}P NMR spectra were measured by using a Bruker AVANCE III (400 MHz) instrument with deuterated DMSO used as the solvent. UV-vis spectroscopy was measured by a UV-vis-NIR 3600 spectrometer (Shimadzu, Japan). XPS data were measured by a THERMO VG ESCALAB 250. FTIR spectra were obtained with a Nicolet 8700 (Thermo Electron Corporation). The XRD patterns were carried out in an X-ray powder diffractometer (XRD-6000, SHIMADZU). SEM spectra was performed with a Hitachi HITACHI S-470. AFM under taping mode were obtained on a Bruker DMFASTSCAN2-SYS. The PL spectra were recorded by a fluorescence spectrophotometer (FLS980, Edinburgh instrument) with an excitation source wavelength of 808 nm. The EIS data were determined under AM1.5G simulated solar radiation by the CHI660E electrochemical workstation. Electroluminescence quantum efficiency was tested by applying external voltage/current sources through the devices (ELCT3010, Enlitech). The TPC measurements were performed by the all-in-one characterization platform Paios developed and commercialized by Fluxim AG, Switzerland. J−V characteristics were recorded using the solar simulator (SS-F5-3A, EnliTech) along with AM 1.5 G irradiation at 100 mW cm^{-2}. The certified standard silicon cell (SRC-2020, EnliTech) was used to calibrate the light intensity. EQE was obtained using the measurement system (QE-R, EnliTech).

DFT calculations

All calculations have been carried out on the basis of ωB97XD/BSI with Gaussian 09 D.01 software package. The BSI donates the mixed basic set, which uses 6-311G(D) for C, H, O, P atoms, and uses LanL2DZ for I and Sn atoms. All structures have been optimized and vibration analysis has been conducted, and it has been found that there is no virtual frequency in the steady state, and only one virtual frequency in the transition state.

Supplementary Figures

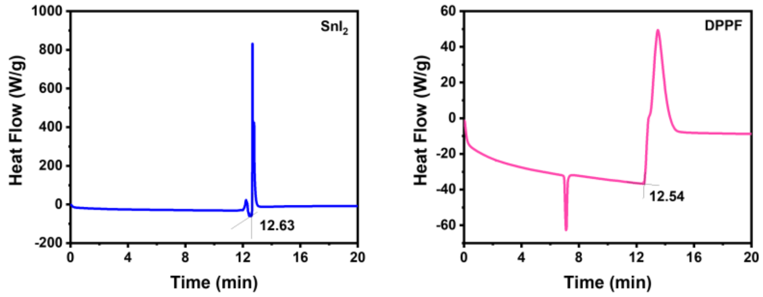

Figure S1. Differential scanning calorimetry curves of SnI_2 and DPPF.

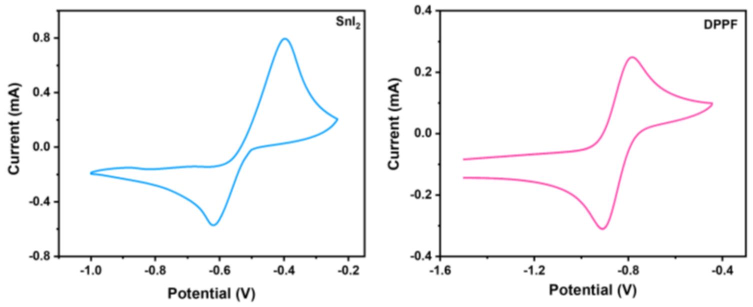

Figure S2. Cyclic voltammetry of SnI_2 and DPPF in DMF.

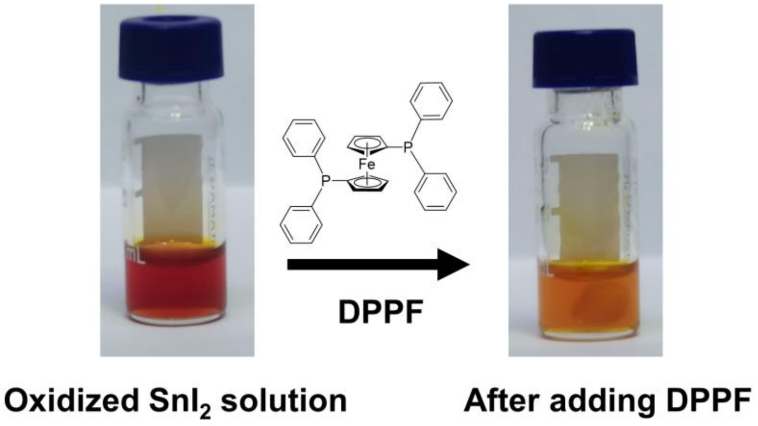

Figure S3. Pictures of the oxidized SnI_2 precursor solution and reduced solution by DPPF additive.

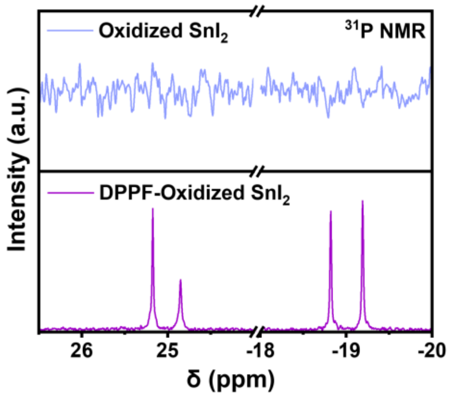

Figure S4. ^{31}P NMR spectra of oxidized SnI_2 and oxidized SnI_2-DPPF.

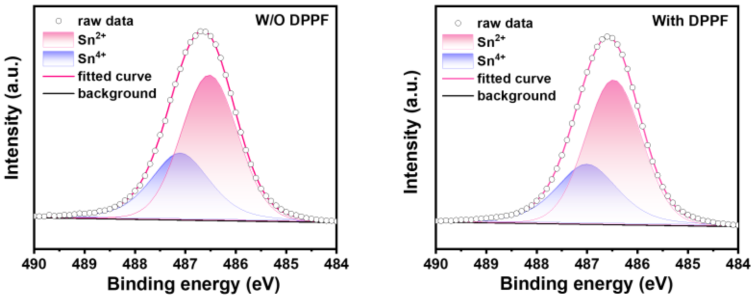

Figure S5. High-resolution XPS Sn 3d spectra of fresh perovskite film without and with DPPF modification.

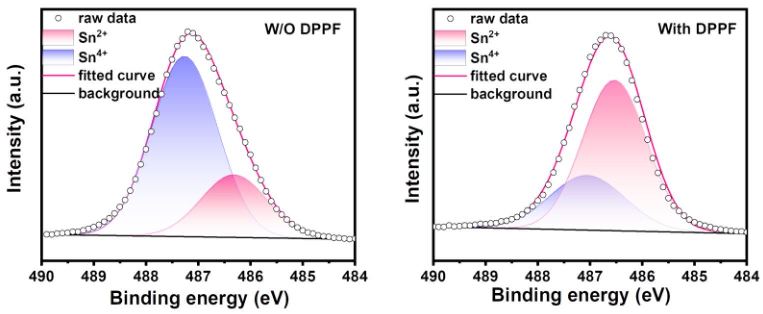

Figure S6. High-resolution XPS Sn 3d spectra of perovskite film without and with DPPF modification.

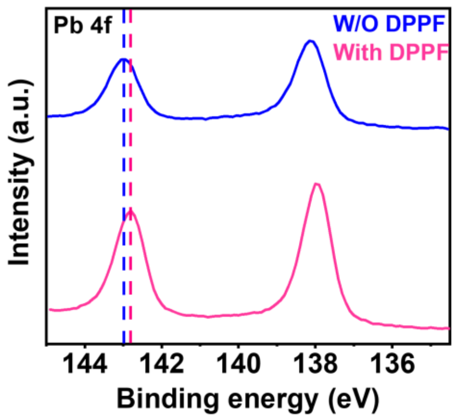

Figure S7. High-resolution XPS Pb 4f spectra of perovskite film without and with DPPF modification.

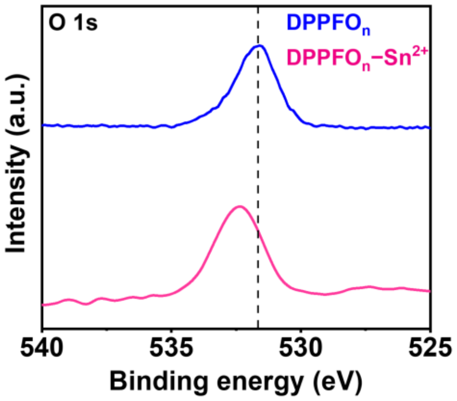

Figure S8. High-resolution XPS O 1s spectra of DPPFO$_n$ and DPPFO$_n$-Sn^{2+}.

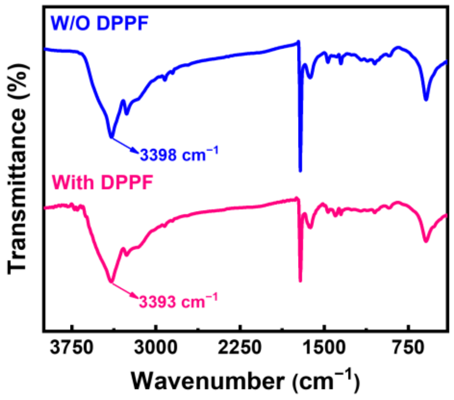

Figure S9. FTIR characterization of perovskite films without and with DPPF modification.

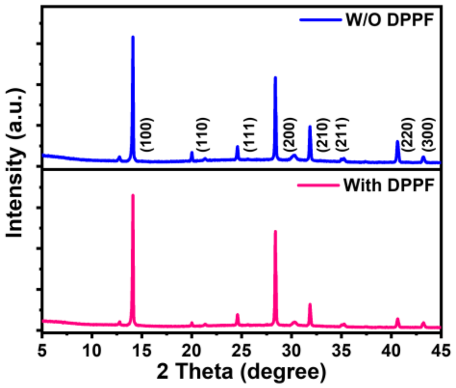

Figure S10. XRD patterns of perovskite films without and with DPPF modification.

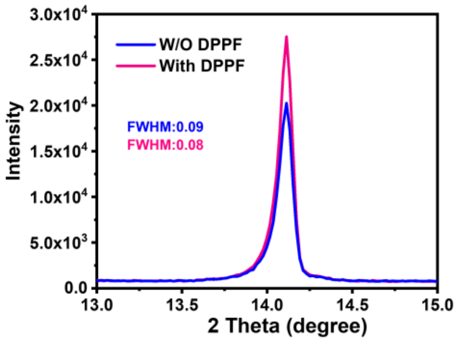

Figure S11. The diffraction intensity and FWHM of (100) peak of perovskite films without and with DPPF modification.

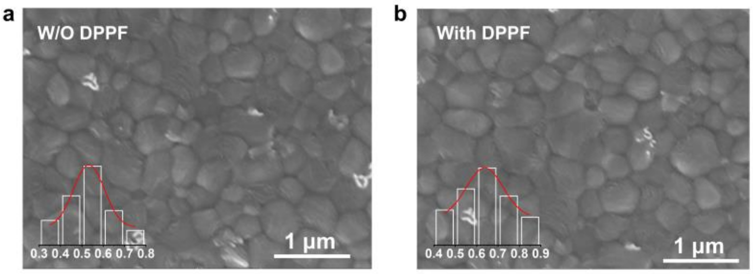

Figure S12. SEM images of perovskite films a) without and b) with DPPF modification. The insets show the histogram of crystal size distribution statistics.

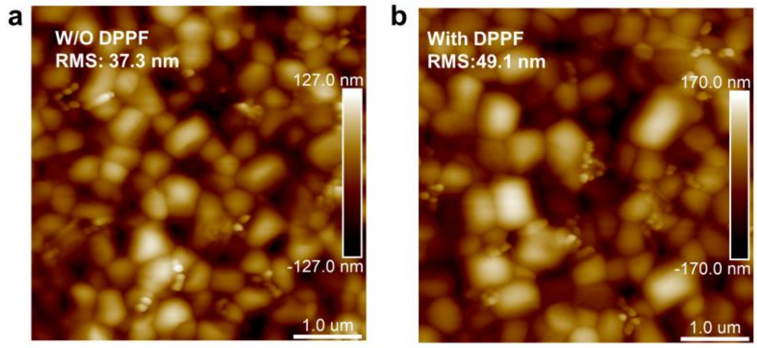

Figure S13. AFM images of perovskite films a) without and b) with DPPF modification.

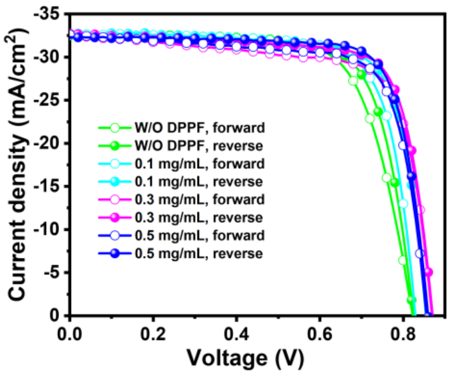

Figure S14. Photovoltaic performance of the device with different concentrations of DPPF (0, 0.1, 0.3 and 0.5 mg/mL).

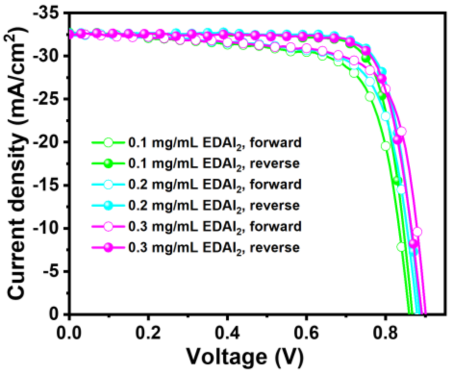

Figure S15. Photovoltaic performances of the devices with different concentrations of $EDAI_2$ (0.1, 0.2 and 0.3 mg/mL).

中国认可
检测
TESTING
CNAS L2338

TEST REPORT

Report No：PWQC-WT-P24041521-1R

**Sample Name**	:	Photovoltaic cell
**Client**	:	Beijing University of Chemical Technology
**Client Address**	:	15 Beisanhuan East Road, Chaoyang District, Beijing, China
**Type of Project**	:	Consignation

PHOTOVOLTAIC AND WIND POWER SYSTEMS QUALITY TEST CENTER. IEE, CHINESE ACADEMY OF SCIENCES

June, 07, 2024

PHOTOVOLTAIC AND WIND POWER SYSTEMS QUALITY TEST CENTER, IEE, CHINESE ACADEMY OF SCIENCES

Report No: PWQC-WT-P24041521-1R

Testing information:

Date：April, 15, 2024

Location：No.6 Bei-er-tiao, Zhongguancun, Haidian district, Beijing, China

Environmental conditions：(24.9±2)℃, (41.4±5)%RH

Testing items：

Measurement of photovoltaic current-voltage characteristics

Standards：

IEC 60904-1：2006 Photovoltaic (PV) devices
— Part 1：Measurement of photovoltaic current-voltage characteristics

Equipments：

Name	S/N	Expired date
Solar simulator	LE106-04	2024-10-15
Source Meter	LE177-01	2025-04-01
Reference cell	J-CH04	2025-04-06

Edited by(signatory)：[illegible]
Date：2024. 6.7

Approved by(signatory)：Jiang Feifei
Date：2024. 6.7

PHOTOVOLTAIC AND WIND POWER SYSTEMS QUALITY TEST CENTER, IEE, CHINESE ACADEMY OF SCIENCES

Report No: PWQC-WT-P24041521-1R

**Sample No.**	DC2024a067
**Sample S/N**	1
**Type**	Single junction perovskite solar cell
**Designated area**	0.02676 cm^2<br>The designated area was certified by National Institute of Metrology, China. Test Report No. CDjc2024-03117.

**Items of testing**	Measurement of photovoltaic current-voltage characteristics					
**Sample code**	DC2024a067					
**Results**	**Voltage Sweep**	**Isc** (mA)	**Jsc** (mA/ cm^2)	**Voc** (V)	**Pm** (mW)	**Curve**
	**Forward**	0.875	32.682	0.886	0.552	A2024041 5092839
		**Ipm** (mA)	**Vpm** (V)	**FF** (%)	**E_{ff}** (%)	
		0.745	0.740	71.15	20.61	
	**Voltage Sweep**	**Isc** (mA)	**Jsc** (mA/ cm^2)	**Voc** (V)	**Pm** (mW)	**Curve**
	**Reverse**	0.873	32.625	0.887	0.626	A2024041 5092842
		**Ipm** (mA)	**Vpm** (V)	**FF** (%)	**E_{ff}** (%)	
		0.823	0.760	80.76	23.38	

Measurement uncertainty:

$U_{95(Isc)}$=1.9% (k=2)

$U_{95(Voc)}$=1.8% (k=2)

$U_{95(Pm)}$=2.5% (k=2)

Page 2 of 4

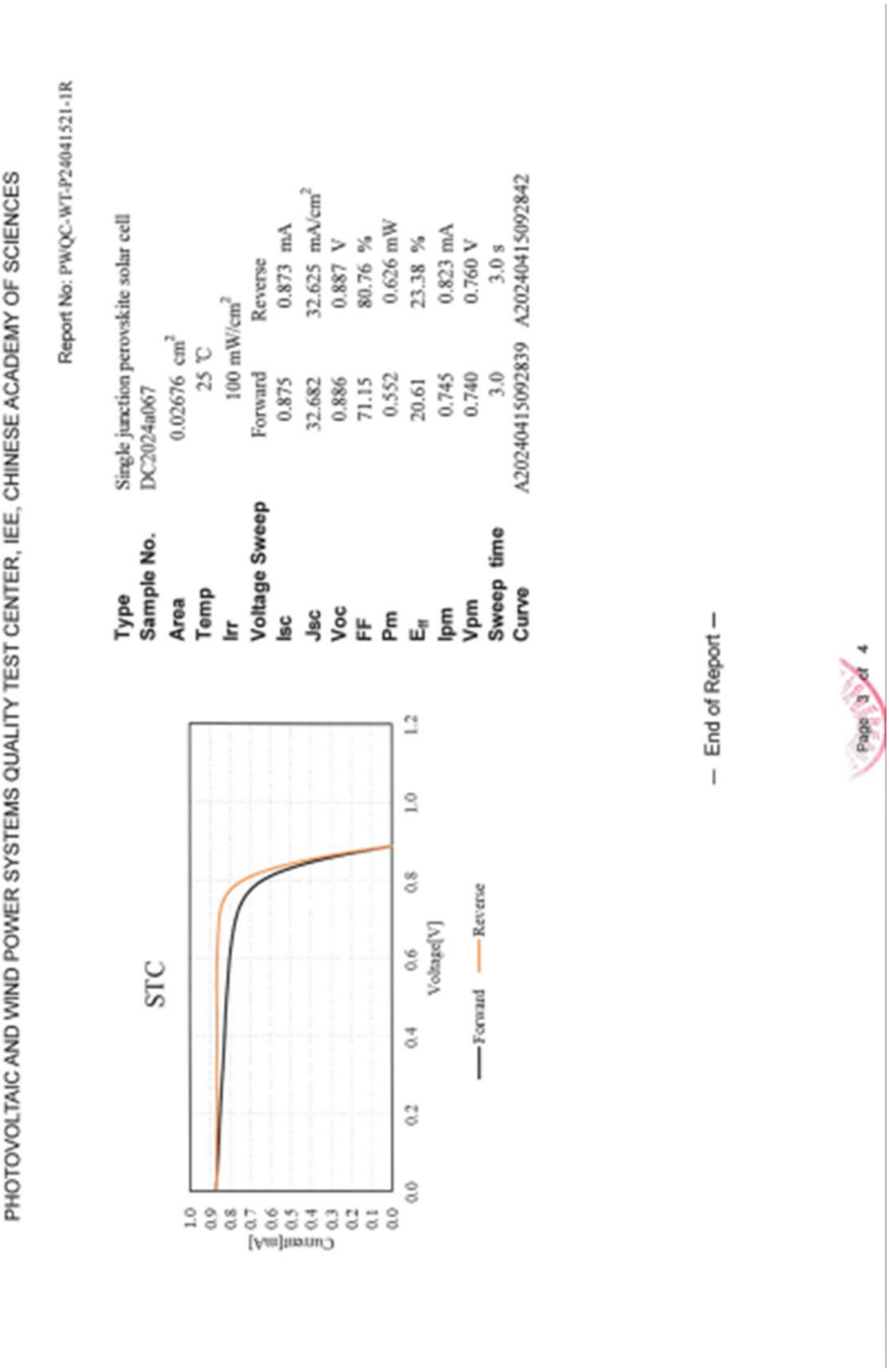
PHOTOVOLTAIC AND WIND POWER SYSTEMS QUALITY TEST CENTER, IEE, CHINESE ACADEMY OF SCIENCES

Report No: PWQC-WT-P24041521-1R

STC

Current[mA]

Voltage[V]

Forward Reverse

Type	Single junction perovskite solar cell	
Sample No.	DC2024a067	
Area	0.02676 cm^2	
Temp	25 ℃	
Irr	100 mW/cm^2	
Voltage Sweep	Forward	Reverse
Isc	0.875	0.873 mA
Jsc	32.682	32.625 mA/cm^2
Voc	0.886	0.887 V
FF	71.15	80.76 %
Pm	0.552	0.626 mW
E_{ff}	20.61	23.38 %
Ipm	0.745	0.823 mA
Vpm	0.740	0.760 V
Sweep time	3.0	3.0 s
Curve	A20240415092839	A20240415092842

— End of Report —

Page 3 of 4

Figure S16. Certified results for Target Sn-Pb PerSC by Institute of Electrical Engineering, Chinese Academy of Sciences. The certified efficiency is 23.38%.

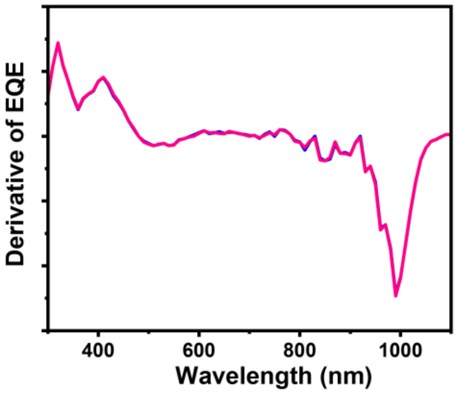

Figure S17. Analysis of perovskite bandgap from the EQE spectrum by taking its derivative spectrum. The bandgap was estimated to be about 1.25 eV.

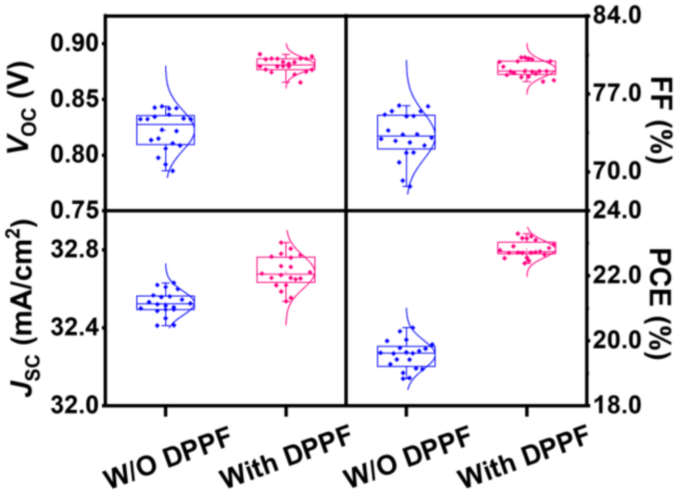

Figure S18. Photovoltaic parameters including V_{OC}, FF, J_{SC}, and PCE of Sn-Pb PerSCs without and with DPPF modification (20 samples for each condition).

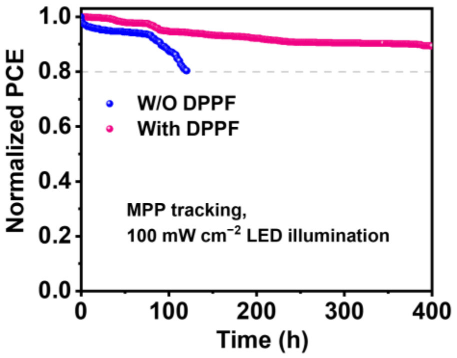

Figure S19. Operational stability under LED illumination of the PerSCs with and without DPPF modification.

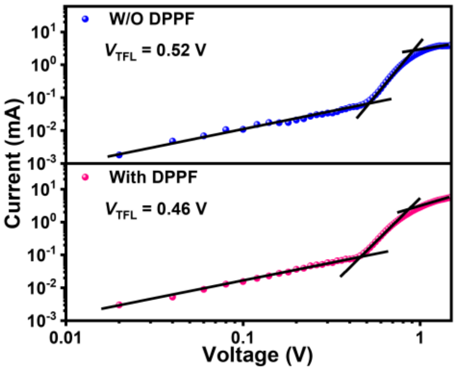

Figure S20. SCLC plots of perovskite films without and with DPPF modification based on a hole-only device (ITO/PEDOT:PSS/perovskite/Spiro-OMeTAD/MoO_3/Ag).

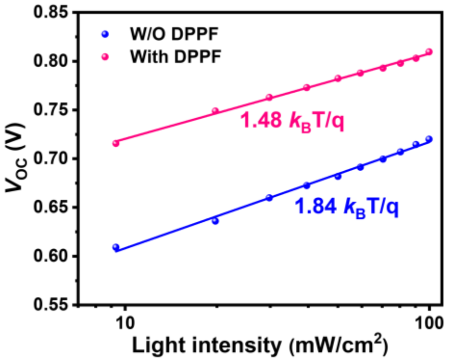

Figure S21. V_{OC} as a function of light intensity for PerSCs without and with DPPF modification.

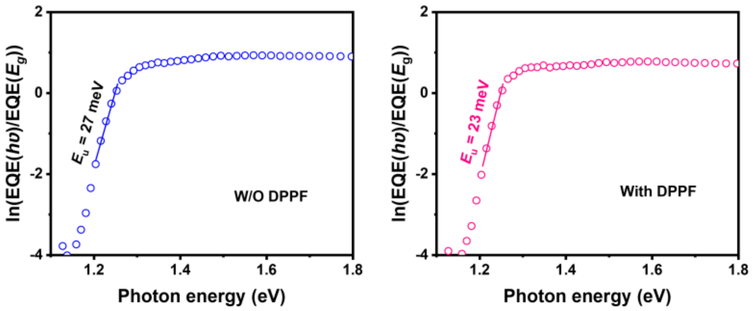

Figure S22. E_u spectra of perovskite films without and with DPPF modification.

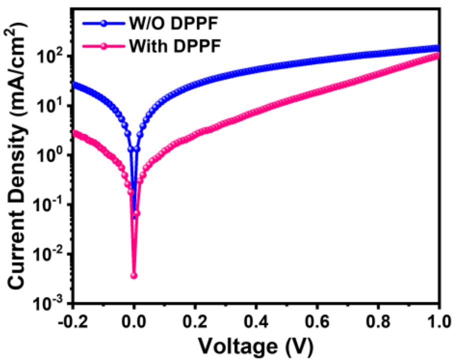

Figure S23. Dark *J*-*V* curves of solar cells without and with DPPF modification.

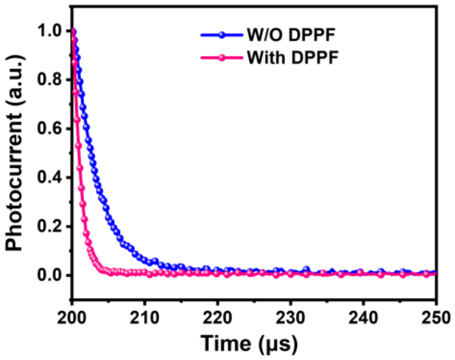

Figure S24. TPC decay curves of the devices based on perovskite films without and with DPPF modification.

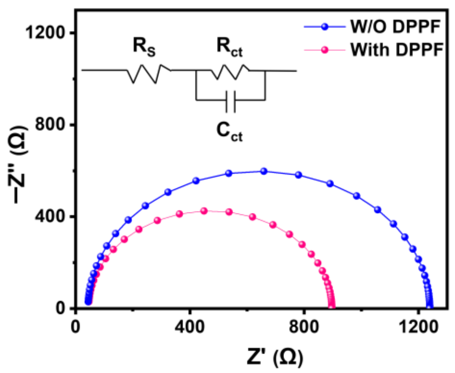

Figure S25. EIS spectra of the Sn-Pb PerSCs without and with DPPF modification. The inset shows the equivalent circuit of PerSCs used for fitting impedance data.

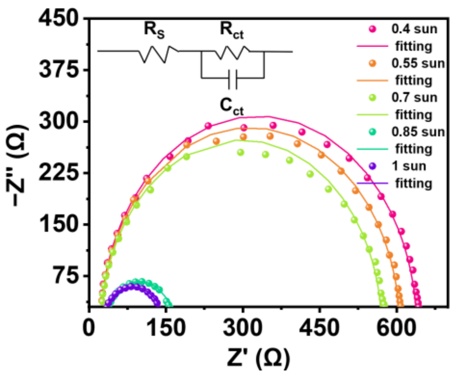

Figure S26. Nyquist plots of PerSCs with DPPF treatment measured in open-circuit conditions for different illumination intensities. The inset shows the equivalent circuit.

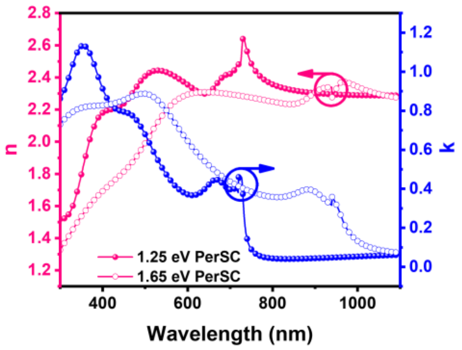

Figure S27. Optical constants of wide-bandgap perovskite and narrow-bandgap perovskite.

Supplementary Tables

Table S1. Summary of photovoltaic parameters of PerSCs incorporated with different concentrations of DPPF additive.

Samples	Scan direction	V_{OC} (V)	FF (%)	J_{SC} (mA cm^{-2})	PCE (%)
W/O DPPF	Forward	0.82	71.1	32.49	18.99
	Reverse	0.82	74.3	32.45	19.86
0.1 mg/mL	Forward	0.83	75.2	32.65	20.36
	Reverse	0.85	75.7	32.77	21.18
0.3 mg/mL	Forward	0.87	72.4	32.73	20.62
	Reverse	0.87	76.2	32.68	21.66
0.5 mg/mL	Forward	0.86	74.0	32.61	20.75
	Reverse	0.86	77.8	32.30	21.61

Table S2. Photovoltaic parameters of PerSCs optimized by different concentrations of $EDAI_2$.

Samples	Scan direction	V_{OC} (V)	FF (%)	J_{SC} (mA cm^{-2})	PCE (%)
0.1 mg/mL	Forward	0.86	72.0	32.66	20.22
	Reverse	0.87	79.3	32.71	22.57
0.2 mg/mL	Forward	0.88	72.9	32.59	20.91
	Reverse	0.89	80.1	32.65	23.28
0.3 mg/mL	Forward	0.89	73.9	32.49	21.37
	Reverse	0.89	79.2	32.54	22.94

Table S3. Electron and hole trap density of perovskite films without and with DPPF modification calculated by SCLC method.

Sample	Structure	V_{TFL} (V)	Trap density (cm^{-3})
W/O DPPF	Electron-only	0.34	1.7×10^{15}
	Hole-only	0.52	2.8×10^{15}
With DPPF	Electron-only	0.23	1.2×10^{15}
	Hole-only	0.46	2.4×10^{15}

Table S4. Summary of photovoltaic parameters and E_{loss} for Sn-Pb PerSCs reported in the literature so far.

Perovskite film	E_g (eV)	V_{OC} (V)	FF (%)	J_{SC} (mA cm^{-2})	PCE (%)	E_{loss} (eV)	Ref.
$Cs_{0.2}FA_{0.8}Pb_{0.5}Sn_{0.5}I_3$	1.29	0.88	80.0	33.80	23.70	0.41	[1]
$FA_{0.7}MA_{0.3}Pb_{0.5}Sn_{0.5}I_3$	1.25	0.88	79.8	31.42	22.16	0.37	[2]
$FA_{0.5}MA_{0.5}Pb_{0.5}Sn_{0.5}I_3$	1.25	0.85	78.0	32.33	21.48	0.40	[3]
$FA_{0.7}MA_{0.27}Cs_{0.03}Pb_{0.5}Sn_{0.5}I_{2.97}Br_{0.03}$	1.25	0.85	80.0	31.14	21.05	0.40	[4]
$FA_{0.7}MA_{0.3}Pb_{0.5}Sn_{0.5}I_3$	1.26	0.83	78.1	30.94	20.03	0.43	[5]
$Cs_{0.05}FA_{0.7}MA_{0.25}Sn_{0.5}Pb_{0.5}I_3$	1.30	0.85	80.6	32.20	22.03	0.45	[6]
$FA_{0.8}MA_{0.2}Pb_{0.8}Sn_{0.2}I_3$	1.35	0.90	81.2	30.88	22.51	0.45	[7]
$FA_{0.7}MA_{0.3}Pb_{0.5}Sn_{0.5}I_3$	1.26	0.86	79.6	29.18	19.85	0.40	[8]
$(FASnI_3)_{0.6}(MAPbI_3)_{0.4}$	1.25	0.83	79.6	30.51	20.20	0.42	[9]
$Cs_{0.025}FA_{0.475}MA_{0.5}Sn_{0.5}Pb_{0.5}I_3$	1.27	0.81	76.0	33.14	20.40	0.46	[10]
$FA_{0.7}MA_{0.3}Pb_{0.5}Sn_{0.5}I_3$	1.25	0.91	77.2	32.99	23.17	0.34	[11]
$Cs_{0.1}FA_{0.6}MA_{0.3}Sn_{0.5}Pb_{0.5}I_3$	1.25	0.89	82.0	32.50	23.60	0.36	[12]
$Cs_{0.3}FA_{0.7}Sn_{0.3}Pb_{0.7}I_3$	1.34	0.79	80.0	29.10	18.30	0.55	[13]
$(MAPbI_3)_{0.75}(FASnI_3)_{0.25}$	1.33	0.78	76.0	28.94	17.16	0.55	[14]
$FA_{0.8}MA_{0.2}Pb_{0.8}Sn_{0.2}I_3$	1.33	0.90	81.2	30.88	22.51	0.43	[15]
$MAPb_{0.75}Sn_{0.25}I_3$	1.30	0.74	79.0	23.5	13.70	0.56	[16]
$Rb_{0.04}Cs_{0.2}FA_{0.76}Pb_{0.5}Sn_{0.5}I_3$	1.25	0.89	80.9	33.42	24.18	0.36	[17]
**$Cs_{0.1}FA_{0.6}MA_{0.3}Sn_{0.5}Pb_{0.5}I_3$**	**1.25**	**0.89**	**80.7**	**32.7**	**23.5**	**0.36**	**This work**

Supplementary Movies

Movie S1. The color change process of SnI_2 solution without and with DPPF.

Movie S2. The color change process of the oxidized SnI_2 solution after adding DPPF.

References

[1] Wang J T, Uddin M A, Chen B *et al.* Enhancing Photostability of Sn-Pb Perovskite Solar Cells by an Alkylammonium Pseudo-Halogen Additive. *Adv Energy Mater* 2023;

[2] Huang L, Cui H, Zhang W *et al.* Efficient Narrow-bandgap Mixed Tin-lead Perovskite Solar Cells via Natural Tin Oxide Doping. *Adv Mater* 2023; e2301125.

[3] Chang Z, Zheng D X, Zhao S *et al.* Designing Heterovalent Substitution with Antioxidant Attribute for High-Performance Sn-Pb Alloyed Perovskite Solar Cells. *Adv Funct Mater* 2023;

[4] Yu D, Wei Q, Li H *et al.* Quasi-2D Bilayer Surface Passivation for High Efficiency Narrow Bandgap Perovskite Solar Cells. *Angew Chem Int Ed Engl* 2022; **61**: e202202346.

[5] Yan N, Ren X D, Fang Z M *et al.* Ligand-Anchoring-Induced Oriented Crystal Growth for High-Efficiency Lead-Tin Perovskite Solar Cells. *Adv Funct Mater* 2022; **32**:

[6] Peng C, Li C, Zhu M *et al.* Reducing Energy Disorder for Efficient and Stable Sn Pb Alloyed Perovskite Solar Cells. *Angew Chem Int Ed Engl* 2022; e202201209.

[7] Liang Z, Xu H, Zhang Y *et al.* A Selective Targeting Anchor Strategy Affords Efficient and Stable Ideal-Bandgap Perovskite Solar Cells. *Adv Mater* 2022; **34**: e2110241.

[8] Liu H, Wang L, Li R *et al.* Modulated Crystallization and Reduced VOC Deficit of Mixed Lead–Tin Perovskite Solar Cells with Antioxidant Caffeic Acid. *ACS Energy Lett* 2021; **6**: 2907-16.

[9] Chen Q, Luo J, He R *et al.* Unveiling Roles of Tin Fluoride Additives in High‐Efficiency Low‐Bandgap Mixed Tin‐Lead Perovskite Solar Cells. *Adv Energy Mater* 2021; **11**:

[10] Kapil G, Bessho T, Ng C H *et al.* Strain Relaxation and Light Management in Tin–Lead Perovskite Solar Cells to Achieve High Efficiencies. *ACS Energy Lett* 2019; **4**: 1991-98.

[11] Zhang W, Yuan H, Li X *et al.* Component Distribution Regulation in Sn-Pb Perovskite Solar Cells through Selective Molecular Interaction. *Adv Mater* 2023; **n/a**: 2303674.

[12] Hu S, Otsuka K, Murdey R *et al.* Optimized carrier extraction at interfaces for 23.6% efficient tin–lead perovskite solar cells. *Energ Environ Sci* 2022; **15**: 2096-107.

[13] Tong J, Gong J, Hu M *et al.* High-performance methylammonium-free ideal-band-gap perovskite solar cells. *Matter* 2021; **4**: 1365-76.

[14] Li C, Pan Y, Hu J *et al.* Vertically Aligned 2D/3D Pb–Sn Perovskites with Enhanced Charge Extraction and Suppressed Phase Segregation for Efficient Printable Solar Cells. *ACS Energy Lett* 2020; **5**: 1386-95.

[15] Liang Z, Xu H, Zhang Y *et al.* A Selective Targeting Anchor Strategy Affords Efficient and Stable Ideal-Bandgap Perovskite Solar Cells. *Adv Mater* 2022; **34**: 2110241.

[16] Liu C, Li W, Li H *et al.* C60 additive-assisted crystallization in CH3NH3Pb0.75Sn0.25I3 perovskite solar cells with high stability and efficiency. *Nanoscale* 2017; **9**: 13967-75.

[17] Zhang Y, Li C, Zhao H *et al*. Synchronized crystallization in tin-lead perovskite solar cells. *Nat Commun* 2024; **15**: 6887.
